# Thermo-Optical Properties of Thin-Film TiO_2_–Al_2_O_3_ Bilayers Fabricated by Atomic Layer Deposition

**DOI:** 10.3390/nano5020792

**Published:** 2015-05-18

**Authors:** Rizwan Ali, Muhammad Rizwan Saleem, Pertti Pääkkönen, Seppo Honkanen

**Affiliations:** 1 Institute of Photonics, University of Eastern Finland, P.O. Box 111, FI-80101 Joensuu, Finland; E-Mails: pertti.paakkonen@uef.fi (P.P.); seppo.honkanen@uef.fi (S.H.); 2 Center for Advance Studies in Energy (CAS-EN), National University of Sciences and Technology (NUST), Sector H-12, 44000 Islamabad, Pakistan

**Keywords:** atomic layer deposition (ALD), thin barrier films, thermo-optic coefficient, titanium dioxide, aluminum oxide, optical materials

## Abstract

We investigate the optical and thermo-optical properties of amorphous TiO_2_–Al_2_O_3_ thin-film bilayers fabricated by atomic layer deposition (ALD). Seven samples of TiO_2_–Al_2_O_3_ bilayers are fabricated by growing Al_2_O_3_ films of different thicknesses on the surface of TiO_2_ films of constant thickness (100 nm). Temperature-induced changes in the optical refractive indices of these thin-film bilayers are measured by a variable angle spectroscopic ellipsometer VASE®. The optical data and the thermo-optic coefficients of the films are retrieved and calculated by applying the Cauchy model and the linear fitting regression algorithm, in order to evaluate the surface porosity model of TiO_2_ films. The effects of TiO_2_ surface defects on the films’ thermo-optic properties are reduced and modified by depositing ultra-thin ALD-Al_2_O_3_ diffusion barrier layers. Increasing the ALD-Al_2_O_3_ thickness from 20 nm to 30 nm results in a sign change of the thermo-optic coefficient of the ALD-TiO_2_. The thermo-optic coefficients of the 100 nm-thick ALD-TiO_2_ film and 30 nm-thick ALD-Al_2_O_3_ film in a bilayer are (0.048 ± 0.134) × 10^−4^ °C^−1^ and (0.680 ± 0.313) × 10^−4^ °C^−1^, respectively, at a temperature *T* = 62 °C.

## 1. Introduction

The continuous miniaturization of optical and photonic components with the growing inquisitiveness to explore atomic-scale phenomena has led researchers to develop innovative techniques to fulfill the requirements for atomic-level control. Atomic layer deposition (ALD), initially developed for the deposition of uniform thin-films over wide areas of electroluminescent display devices [1], is a unique method enabling an atomic-level control of film thickness and reproducible growth of defect-free films. It is cost effective and, thus, suitable for low-cost mass production. Research and development in ALD during recent years has led to a wide range of processes for new materials. In addition, the availability of ALD reactors has paved the way for further improving the precise atomic-level thickness control, achieving self-controlled alternating surface reactions, improving the wide area uniformity and obtaining continuous pinhole or defect-free films [2]. With ALD, high quality thin-films can be deposited even at room temperature [3], but also at high temperatures (up to 500 °C) [4]. One important feature of ALD is the excellent conformality of the deposited films over corrugated and/or zigzag surface profiles [5].

The ALD process is based on sequential self-limiting chemical reactions of alternating precursor molecules on the substrate surface at a given deposition temperature, which results in saturative surface reactions. A typical ALD process proceeds in cycles composed of four steps. During the first step, precursor “A” molecules are pulsed into the ALD chamber to chemisorb on the substrate surface followed by purging with an inert gas to remove excessive precursor molecules or any other reaction by-products (the second step). In the third step, the ALD chamber is pulsed with precursor “B” molecules, which react with precursor “A” molecules chemisorbed on the substrate surface and form the desired binary compound. This step is followed again by purging with an inert gas to remove non-reactive precursor “B” molecules and/or any reaction by-products (the fourth step). This one complete AB ALD cycle results in the deposition of a partial monolayer, typically with a thickness of around 0.1 nm/cycle. Several ABAB ALD cycles are needed to form a uniform and high-quality film [6,7].

Titanium dioxide (TiO_2_) is a widely-studied metal oxide material due to having a high relative dielectric constant, high melting point, stability at higher temperatures, low absorption in the visible and infrared wavelength regions and temperature-induced reflectivity [8,9]. ALD-deposited TiO_2_ has significant potential in nanophotonics applications [6]. An important aspect of ALD-deposited TiO_2_ films is the adsorption of water molecules during the growth process, which leads to a change in the refractive index of the films due to environmental effects, such as temperature, pressure and humidity. Such index changes can also be accompanied by a bandgap change of the dielectric films due to the adsorption of water or other hydrogenated species at interstitial positions [10].

**Figure 1.**
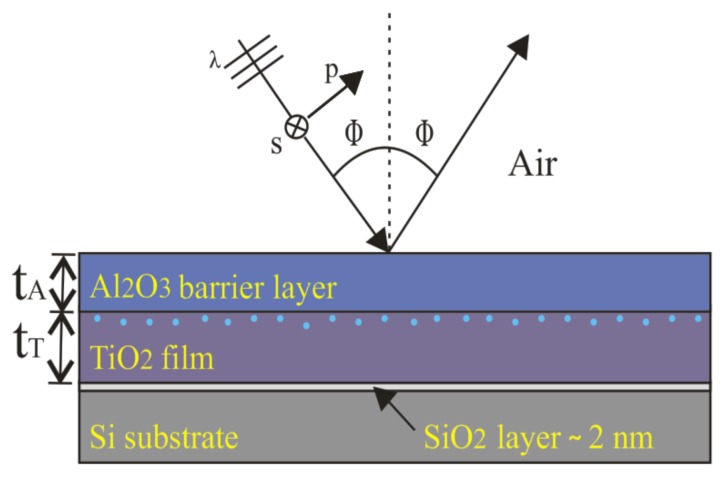
Schematic diagram of a TiO_2_ and Al_2_O_3_ bilayer film grown by atomic layer deposition (ALD) on a Si substrate. The illumination geometry in ellipsometric measurements is also shown.

In this paper, we propose to modify the thermo-optic coefficient of TiO_2_ thin-films by employing a TiO_2_–Al_2_O_3_ bilayer film (see Figure 1). This may be useful in various nanophotonic devices, e.g., if athermal optical properties are desired. Due to the excellent film uniformity and low surface roughness obtained by ALD, it is an ideal deposition method for the fabrication of TiO_2_–Al_2_O_3_ bilayers [11]. We study the thermo-optic coefficients of ALD-TiO_2_ films of a 100-nm thickness and the influence of the ALD-Al_2_O_3_ thin-films of different thicknesses (10–70 nm) as barrier layers, in order to validate the surface porosity model introduced earlier [10,12]. The proposed model explains the reduction of surface defects on TiO_2_ films due to the ALD-Al_2_O_3_ diffusion barrier layer and the reduction of the evaporation rate of water molecules from TiO_2_ films through the ALD-Al_2_O_3_ diffusion barrier layers. The concept is based on the assumption that the TiO_2_ surface maintains the same energy levels (bandgaps) after coating by ultra-thin ALD-Al_2_O_3_ diffusion barrier layers. This is because the interfacial energy levels may change significantly due to the evaporation rate of water molecules from the TiO_2_ surface and/or chemical reactions or environmental interactions.

The paper is organized as follows: Section 2 describes the fabrication, thermal measurements and the formulated optical model viable for the evaluation of ellipsometric parameters and stacked layer thicknesses through a polynomial fitting. Section 3 presents the experimental results and analysis of TiO_2_ films of 100-nm thickness coated by ALD-Al_2_O_3_ diffusion barrier layers with different thicknesses. Finally, conclusions are presented in Section 4.

## 2. Experiments and Data Analysis

### 2.1. Sample Fabrication

In this study, seven bilayer samples containing a 100 nm-thick amorphous TiO_2_ film and amorphous Al_2_O_3_ diffusion barrier layers with different thicknesses (from 10 nm to 70 nm) were fabricated by ALD on 2-inch silicon wafers with crystal orientation <100> by using a Beneq TFS 200-152 ALD reactor. The samples were not exposed to ambient environment between the depositions of the two layers. The employed wafers were doped with phosphorus (n-type), have a thickness of 380 ± 25 μm and a native oxide layer with a thickness of 2 nm. The thickness of each ALD-grown layer was measured by the Dektak-150 stylus surface profilometer from Veeco technology, and the values were confirmed by ellipsometry measurements. The precursor species for the deposition of TiO_2_ and Al_2_O_3_ thin-films were titanium tetrachloride (TiCl_4_) and trimethylaluminum (Al(CH_3_)_3_), respectively, as well as H_2_O. Nitrogen (N_2_) was used as a purging gas. During the processes, the ALD chamber and the reactor were maintained at pressures of 5 mbar and 2 mbar, respectively. The ALD chamber N_2_ flow was 200 sccm (standard cubic centimeters per minute), and the process N_2_ flow was 600 sccm. During the TiO_2_ film deposition, the TiCl_4_ pulse time was 150 ms, and the N_2_ purge time was 750 ms; whereas the H_2_O pulse time was 150 ms, and the N_2_ purge time was 1 s. After the deposition process, the temperature was set to 20 °C, and the gas ballast time was 5 min to stabilize the end of the deposition process. At the end of the deposition process, the TiCl_4_ gas line was purged with N_2_ gas for 2 min. TiO_2_ thin-film deposition was carried out at a temperature of 120 °C. Except for the Al precursor, the same deposition cycle parameters were used to deposit the Al_2_O_3_ thin-films. The growth rates of TiO_2_ and Al_2_O_3_ thin-films were 0.065 nm and 0.12 nm per cycle, respectively.

### 2.2. Thermal Measurement Setup

The thickness-, wavelength- and temperature-dependent refractive indices *n* ≡ *n*(*t*_T,A_, λ, *T*) of the bilayer TiO_2_ and Al_2_O_3_ thin-films were characterized by a variable angle spectroscopic ellipsometer VASE® with a High-Speed Monochromator System HS-190TM (manufactured by J.A. Woollam Co., Lincoln, NE, USA). It contains a continuously-rotating and adjustable wave plate analyzer, and a maximum beam spot size of 3 mm was used. The temperature-dependent refractive index of each bilayer sample was measured by a customized temperature-controlled heating device fabricated in-house. The heating device consists of an aluminum plate with adjustable mechanical clampers to hold the sample, thermocouples, a thermometer (manufactured by Fluke Corporation, Everett, WA, USA) and an adjustable power supply. The measurements were carried out for a temperature range from *T* = 22 °C to *T* = 102 °C with temperature steps of 10 °C and with a reading accuracy of ±0.1 °C. The temperature was precisely controlled by the adjustable power supply, and it was also measured by a Handheld Infrared Thermometer (Model Convir ST8811, manufactured by Calex Electronics Limited Co., Bedfordshire, UK). The measurements were done for a spectral range from 380 nm to 1800 nm with 5 nm wavelength steps. Three angles of incidence (59°, 67° and 75°) around the pseudo-Brewster angle were used in measurements for each temperature.

### 2.3. Structural Measurements

For structural characterization of the TiO_2_ and Al_2_O_3_ thin-film bilayers, the samples were sputter coated with a thin (less than 8 nm thick) conductive copper (Cu) by a K675X Turbo Large Chromium Coater 8. The sputter-coated samples were characterized by a scanning electron microscope (SEM), LEO 1550 Gemini. The surface morphology measurements of the ALD-TiO_2_–Al_2_O_3_ thin-film bilayers were carried out by an atomic force microscope (AFM, MultiMode® with High-Speed ScanAsyst-Air Mode, Bruker Co., Billerica, MA, USA). The tapping mode in air was employed, and the software Nanoscope Analysis Version 1.50 was utilized in the AFM image analysis. The surface roughness of each sample was measured by scanning a surface area of 1 μm × 1 μm of the sample.

### 2.4. Ellipsometry and Optical Model for Bilayer

As mentioned above, a variable angle spectroscopic ellipsometer was used to characterize and determine the optical properties of the fabricated samples. The analysis was carried out with WVASE32 ellipsometric data analysis software from J.A. Woollam Co., in terms of ellipsometric parameters Ψ and Δ to express the polarization state of a reflected light beam [12,13]. Figure 1 illustrates schematically the interaction of polarized light with a sample and also the porosity at the surface of the TiO_2_ layer. The varying angles of incidence in spectroscopic ellipsometry were set near the pseudo-Brewster angle to get the best sensitivity for the measured values of Δ [14].

In our optical model (see Figure 1), a polarized light (plane wave) illuminates a bilayer at an angle of incidence Φ. In this model, a 100 nm-thick (*t*_T_) TiO_2_ film is considered on a native oxide (SiO_2_) layer of ~2 nm in thickness (confirmed also with ellipsometry measurements). This, in turn, is on top of a 0.5 mm-thick Si substrate. On top of the TiO_2_ film, a thin layer of Al_2_O_3_ is added. Its thickness *t*_A_ is varied from 10 nm to 70 nm. The model usesWVASE32 ellipsometry software in fitting the ellipsometric parameters Ψ and Δ to retrieve the experimentally-measured data at each wavelength within the defined spectral range. We did not add the layer of surface roughness and voids to the model (see Figure 1), since it would have been very difficult to properly add this non-uniform layer to the model. In addition, this layer would have a negligible effect, since the average surface roughness values of the TiO_2_ and Al_2_O_3_ nanoscale layers are only ≈1 nm. To assure the validity of this optical model, the errors were computed by a regressive algorithm and identified within a 90% confidence limit.

#### 2.4.1. Retrieval of the Film Thickness and Optical Constants by Optical Models

The thicknesses and optical constants of TiO_2_ and Al_2_O_3_ in the fabricated thin bilayer films were extracted by a Cauchy model. The optical constants of the films in these bilayers, with a corresponding uncertainty (statistical deviation values), were calculated at a wavelength of 640 nm. The Cauchy dispersion formula and the corresponding uncertainty equations used are given below, respectively, in Equations (1) and (2) [15]:

(1) $$n(\lambda )=A+\frac{B}{\lambda^{2}}+\frac{C}{\lambda^{4}}$$

(2) $$\sigma [n(\lambda )]=[{\left(\sigma A\right)}^{2}+{\left(\frac{\sigma B}{\lambda^{2}}\right)}^{2}+{\left(\frac{\sigma C}{\lambda^{4}}\right)}^{2}]$$

Here, *A*, *B* and *C* are Cauchy parameters and the unit of wavelength is μm. The final values of Cauchy model parameters were determined by fitting the model-generated data to the experimental data.

**Figure 2.**
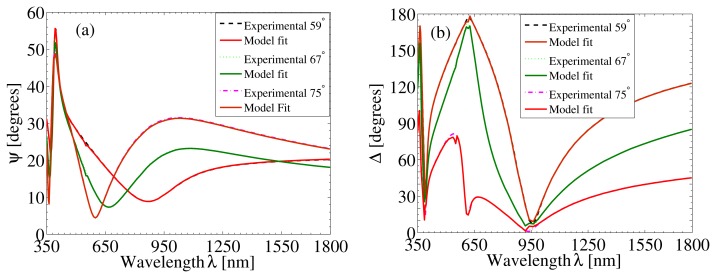
Spectral dependence of the measured ellipsometric data at room temperature: (**a**) Ψ and (**b**) Δ for a bilayer with a 100 nm-thick ALD-TiO_2_ and a 30 nm-thick ALD-Al_2_O_3_. The data are fitted by the Cauchy model at three different angles (59°, 67° and 75°).

Thermal measurements for the bare Si substrate, with a thin native oxide layer, were first carried out for the temperature range from *T* = 22 °C to *T* = 102 °C, as described in Section 2.2. The thermal results for the Si substrate were added as the default for each temperature point in the beginning of the modeling scheme. The thermal data for the SiO_2_ layer embedded in the WVASE32 software was used. The appropriate minimum and maximum thickness values of each layer in the bilayer, together with the initial values of Cauchy parameters, were assigned using the edit fitting parameters. Upon optimum fitting between the experimental and the model data, the thickness and optical constants of each layer in the bilayer were determined, at a wavelength of 640 nm. Figure 2 shows room temperature spectroscopic ellipsometric measurements, in terms of ellipsometric parameters Ψ and Δ fitted by the Cauchy model at three angles (59°, 67° and 75°), for a bilayer consisting of a 100 nm-thick TiO_2_ layer and a 30 nm-thick Al_2_O_3_ layer.

#### 2.4.2. Polynomial Fitting by Regression Algorithm

Finally, the thermo-optic coefficients for each layer in the bilayer along with their uncertainties were calculated using the refractive index data calculated at a 640 nm wavelength and employing a polynomial fitting algorithm. The procedure of this polynomial fitting is explained in detail in [16,17]. In our case, the thermo-optic coefficients and the associated standard uncertainties are at the 90% level of confidence.

## 3. Results and Discussion

**Figure 3.**
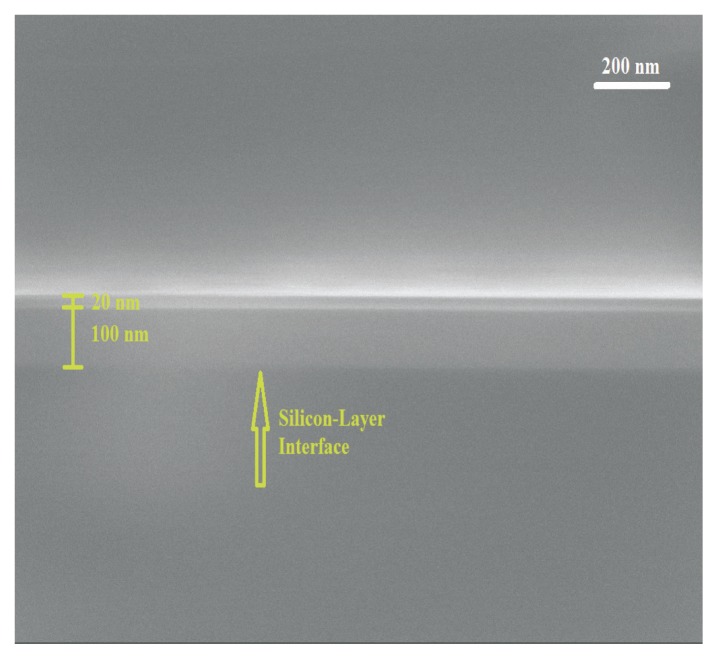
Scanning electron microscope (SEM) image of a bilayer consisting of a 100 nm-thick ALD-TiO_2_ and a 20 nm-thick ALD-Al_2_O_3_ film, on a Si substrate.

Thermo-optic coefficients of the bilayers comprising a 100 nm-thick ALD-TiO_2_ film and ALD-Al_2_O_3_ films of different thicknesses, from 10 nm to 70 nm, were evaluated after fitting the experimentally-measured data. To illustrate the high quality of these bilayers, Figure 3 shows an SEM image of a cross-section of a bilayer with 20 nm-thick ALD-Al_2_O_3_ diffusion barrier layer to protect the TiO_2_ surface from water vapor evaporation.

**Figure 4.**
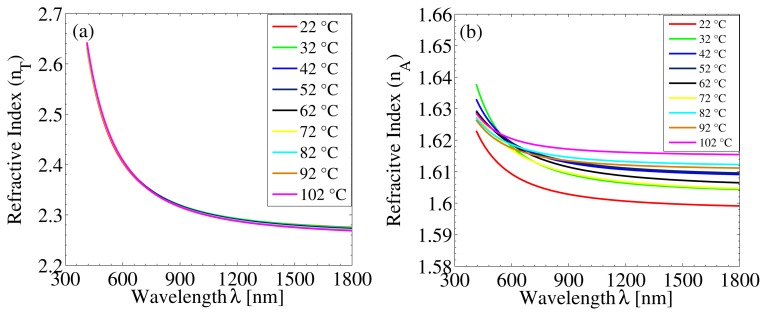
Dispersion relation of refractive indices of (**a**) a 100 nm-thick ALD-TiO_2_ film and (**b**) a 30 nm-thick ALD-Al_2_O_3_ film in a TiO_2_–Al_2_O_3_ bilayer at different temperatures.

It has been previously reported that TiO_2_ films possess negative thermo-optic coefficients due to the desorption of H_2_O and OH^−^ species at the OH–H_2_O surface-bound sites [12,18,19]. The adsorption of water molecules either undergoes dissociations at particular defect sites or they are adsorbed molecularly. Such evaporation of water molecules can be effectively minimized by an ALD-deposited Al_2_O_3_ diffusion barrier layer. This is due to the pinhole and defect-free nature of even very thin ALD-Al_2_O_3_ films, which cap the TiO_2_ surface and eliminate the desorption of water molecules at elevated temperatures. Figure 4a shows the temperature dependence of the refractive index dispersion curves of a 100 nm-thick ALD-TiO_2_ film coated by a 30 nm-thick ALD-Al_2_O_3_ diffusion barrier layer. Figure 4b shows the same for the 30 nm-thick Al_2_O_3_ barrier layer. Considering the calculation of thermo-optic coefficients at the wavelength of 640 nm, one can observe that the ALD-TiO_2_ index is nearly constant, and the Al_2_O_3_ index increases with temperature [20].

**Figure 5.**
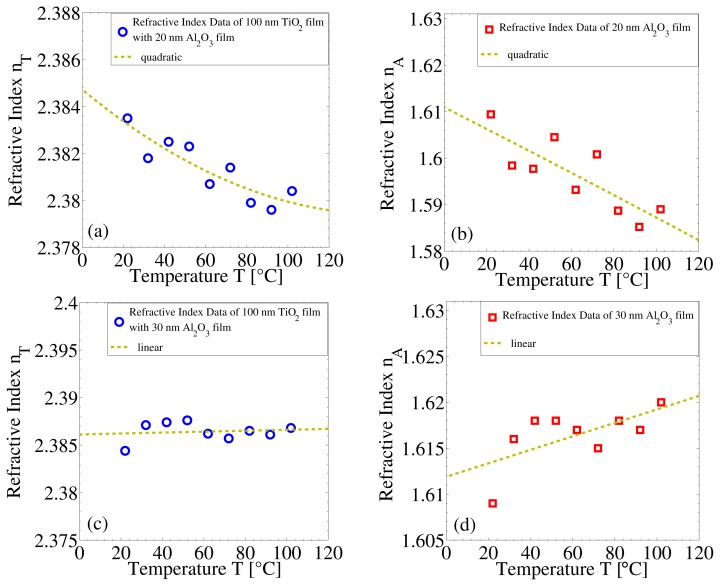
Temperature dependence of the refractive index of ALD-TiO_2_ and ALD-Al_2_O_3_ films in bilayers: (**a**) for a 100 nm-thick TiO_2_ film with a 20 nm-thick Al_2_O_3_ diffusion barrier layer; (**b**) for a 20-nm Al_2_O_3_ diffusion barrier layer; (**c**) for a 100-nm TiO_2_ film with a 30-nm Al_2_O_3_ diffusion barrier layer; and (**d**) for a 30-nm Al_2_O_3_ diffusion barrier layer.

The temperature dependence of the refractive index of the 100 nm-thick ALD-TiO_2_ film as a function of the Al_2_O_3_ film thickness, *n*_T_(*T*, *t*_A_), at a wavelength of 640 nm, is shown in Figure 5. This TiO_2_ temperature dependence is shown in the case of the ALD-Al_2_O_3_ diffusion barrier layer thicknesses of 20 nm (Figure 5a) and 30 nm (Figure 5c). The corresponding temperature dependencies of the Al_2_O_3_ diffusion barrier layers are shown in Figure 5b,d. As mentioned above, with a 20 nm-thick ALD-Al_2_O_3_ diffusion barrier layer, the refractive index of the 100 nm-thick ALD-TiO_2_ film decreases slightly with temperature. This temperature dependence is very similar to that of a single 100 nm-thick TiO_2_ film, indicating a very small barrier effect [12]. However, by increasing the diffusion barrier layer thickness only by 10 nm, *i.e.*, to 30 nm, the refractive index of the TiO_2_ is nearly constant with temperature. It can also be seen (Figure 5d) that the refractive index of the 30 nm-thick ALD-Al_2_O_3_ film increases with temperature rather than decreases, as is the case with the 20 nm-thick ALD-Al_2_O_3_ film (Figure 5b). This is expected when the Al_2_O_3_ film is thick enough. These results clearly indicate that a 20 nm-thick ALD-Al_2_O_3_ film is not yet pinhole free, while a 30 nm-thick film acts as an efficient, nearly pinhole-free, diffusion barrier layer. Consequently, a different behavior of the refractive indices with the temperature is observed for the ALD-Al_2_O_3_ films of 20-nm and 30-nm thicknesses in Figure 5b,d, respectively. In addition, the temperature dependence of the refractive index of a TiO_2_ thin-film does not depend only on its own properties, but also on the thickness of the diffusion barrier layer.

**Figure 6.**
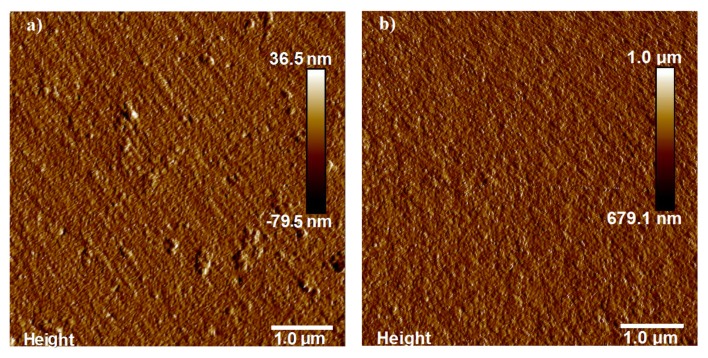
The atomic force microscopy (AFM) images of (**a**) a 100 nm-thick ALD-TiO_2_ film deposited on a Si substrate with a surface roughness value *R_a_* = 1.10 nm; and (**b**) a 100 nm-thick ALD-TiO_2_ film with a 20 nm-thick ALD-Al_2_O_3_ film with a surface roughness value *R_a_* < 1 nm.

Figure 6a shows an atomic force microscopy (AFM) image of a 100 nm-thick ALD-TiO_2_ film with a surface roughness of *R_a_* = 1.10 nm. The surface roughness of a similar 100 nm-thick ALD-TiO_2_ film with a 20 nm-thick ALD-Al_2_O_3_ film is less than 1 nm and is shown in Figure 6b. This means that the surface roughness of the TiO_2_ films is reduced after coating with an ALD-Al_2_O_3_ film.

The temperature dependence of the permeation through a diffusion barrier layer provides information about the dominating permeation mechanism for H_2_O and OH^−^ species, since the vapor permeation through solids is a thermally-driven process. The temperature-dependent permeation process can be described by the Arrhenius equation [21] as:

(3) $$q=q_{0}.e^{\frac{-E_{\text{A}}}{kT}}$$

where *q* is the permeation coefficient, *q*_0_ is the system-dependent constant permeation coefficient, *E*_A_ is the activation energy and *k* is the Boltzmann constant. The activation energies depend on the diffusion barrier layer, which is interpreted by a chemical interaction between water molecules and the coated diffusion barrier layer.

**Figure 7.**
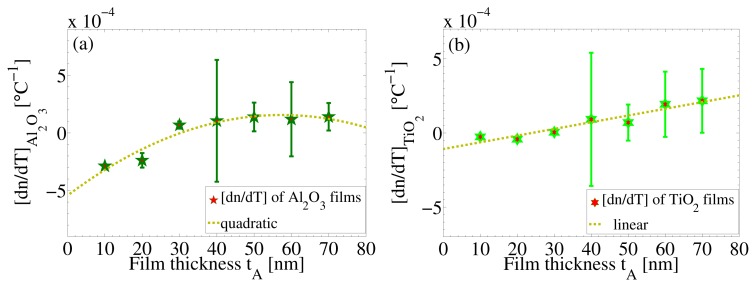
Thermo-optic coefficients of (**a**) Al_2_O_3_ and (**b**) TiO_2_ films.

Figure 7 shows the thermo-optic coefficients of ALD-Al_2_O_3_ and ALD-TiO_2_ films as a function of the thickness *t*_A_. The thermo-optic coefficient of the Al_2_O_3_ film attains a positive value when the thickness *t*_A_ is 30 nm or more (Figure 7a), *i.e.*, when the film is nearly pinhole free and uniform. With thicker films, up to 70 nm in thickness, no rapid increase in the thermo-optic coefficient appears. On the other hand, the thermo-optic coefficient of the 100 nm-thick ALD-TiO_2_ film appears to increase linearly with thicknesses *t*_A_, as seen in Figure 7b. A linear fit gives an equation:

(4) $${\left[\frac{\text{d}n}{\text{d}T}\right]}_{{\text{TiO}}_{2}}=6.7\times 10^{-6}t_{\text{A}}-2.2\times 10^{-4}$$

For its dependence on *t*_A_. Here, the slope of 6.7 × 10^−6^ has dimensions of °C^−1^ nm^−1^; −2.2 × 10^−4^ is a constant with a dimension of °C^−1^; and *t*_A_ is the thickness of the barrier layer in nm. The slope of the straight line is described as per degree increase in temperature per nm, which determines the amount of activation energy required for water molecules to permeate through the ALD-Al_2_O_3_ diffusion barrier layer. If one increases the thickness *t*_A_ of the barrier layer, the required activation energy will increase, and less water molecules are able to permeate through the barrier layers. This, in turn, will result in an increase in the thermo-optic coefficient of the TiO_2_ film and, consequently, in an increase in its density. Interestingly, the thermo-optic coefficient can attain a positive value, although the TiO_2_ films typically possess a negative thermo-optic coefficient. This may also be interpreted as acquiring low thermodynamic energies in order to fill surface defects of TiO_2_ films to minimize pinhole defects. As a potential application for these ALD-TiO_2_–Al_2_O_3_ bilayer films and nanostacks, we propose resonance waveguide gratings (RWG), which have important applications in sensing [22].

**Figure 8.**
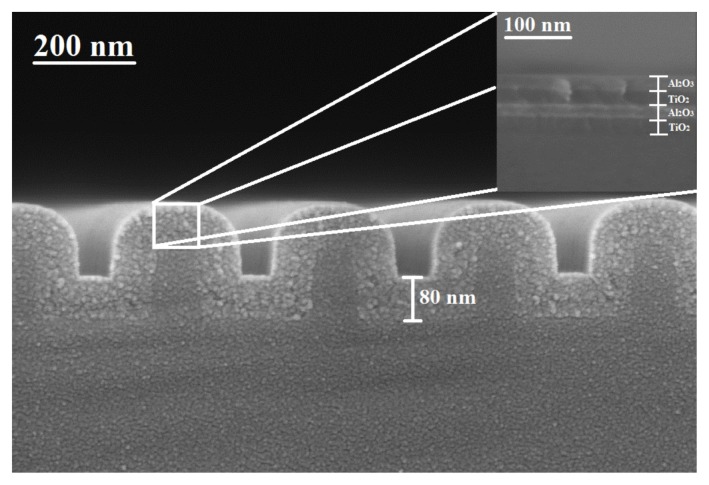
SEM image of a resonant waveguide grating structure with a TiO_2_–Al_2_O_3_ bilayer as the waveguide layer, on a fused silica substrate.

Typically, the RWG spectral characteristics encounter a spectral shift under varying thermal conditions and consequently make the resonance peak unstable for accurate sensing [23]. In order to mitigate the effect of temperature-dependent variations in the refractive index of TiO_2_, RWG surfaces can be coated with thin Al_2_O_3_ layers as diffusion barrier layers. To illustrate this approach, Figure 8 shows an RWG structure fabricated on fused silica substrate and employing an ALD-TiO_2_–Al_2_O_3_ nanostack as the waveguide. It is worth mentioning that the use of ALD results in highly conformal coating on the corrugated substrate.

## 4. Conclusions

Thin-film bilayers of TiO_2_ and Al_2_O_3_ were fabricated by ALD, and their temperature-dependent optical properties were spectrally characterized by ellipsometry. The experimental data were analyzed with the support of optical models and respective regression algorithms. The ellipsometry data measured for these bilayers were fitted by the Cauchy model, which provided optical constants both for TiO_2_ and Al_2_O_3_ thin-films. The determined optical constants were then used for the evaluation of thermo-optic constants of TiO_2_ and Al_2_O_3_ thin-films in this bilayer configuration. Thermo-optic properties of ALD-TiO_2_ films with a thickness of 100 nm, in the presence of diffusion barrier layers of ALD-Al_2_O_3_ with different thicknesses, were presented. The results were discussed in terms of the porosity model of thin TiO_2_ films. The study included the gradual increase of the thickness of the diffusion barrier layer, which resulted in a change in the refractive index and thermo-optic coefficients of the two thin-film materials. On increasing the thickness of the ALD-Al_2_O_3_ diffusion barrier layers from 20 nm to 30 nm, the initially negative thermo-optic coefficient of a 100 nm-thick TiO_2_ film turns positive. The increase in its thermo-optic coefficient with the thickness of the barrier layer exhibits a nearly linear relation, with a slope that determines the activation energies required for H_2_O and OH^−^ species to permeate through the barrier layer. The proposed ALD-grown bilayers and nanostacks may have potential in nanophotonics applications, in which temperature-dependent operation is a nuisance. For example, biosensors based on resonance waveguide gratings typically require accurate control of the ambient temperature, which may be alleviated with the proposed approach.
